# Milk Sickness

**Published:** 1860-03

**Authors:** 


***Milk Sickness.***

In the *Nashville Journal of Medicine and Surgery*, October, J. E. Nagle, M. D., has an article upon the above subject. He considers the disease a peculiar form of typhus fever, and he supposes it to be produced by a poisonous fungus grass. He says, "that musty wheat and secale cornutum of rye will produce the gangrene typhus fever, is evidenced on good authority; that the fungus of grass has the same poisonous principle I am certain and I think will be admitted if the subject be examined."

With commendable zeal Dr. Nagle experimented largely. On lands upon which this fungus grass was largely found, he pastured certain animals. "In a short time we discovered the disease was making way amongst them; those that died were subjected to post-mortem examination, with the result above stated. We then cut the grass and cured it as hay, which we fed to healthy, fresh animals. They soon gave evidence of distress: became thin in flesh, had excessive heat in the head and neck, ashriveled skin over the belly, were exceedingly costive, and giving way to delirium and debility, they died in utter exhaustion and agony. We then found that dogs fed upon the meat of such animals would get the same 'trembles,' the same symptoms, the same death, the same poisoned appearance on examination." * * "Not satisfied, however, we pushed the inquiry further, and discovered that this grass, when fed alone without mixture, produced the most horrible typhus fever—a pure poisonous fever, that was milk sickness in all its hydra gangrenous condition." * * "The result to cattle becoming thus diseased gives rise to sickness in the human body. The poison tends to the glands, and the secretion of milk will necessarily be charged with it; hence, when an animal uses the milk, or eats the flesh of such diseased cattle, the result must be a similar disease in the recipient."

There are but few subjects in medicine that have occasioned more controversy than the disease under consideration, and we look upon the paper of Dr. Nagle as one of the most satisfactory that has fallen under our observation. By many, milk sickness has been considered a modification of malarious disease, and by others it has been supposed to be produced by some unknown toxic agent. Attempts have been made to specify the peculiar poison, but none, so far as we know, have based their opinions upon such careful and extended observation as has Dr. Nagle.

We quote his closing remarks in regard to treatment. "The main object is to keep up tonic, stimulant treatment, and particularly to keep the bowels open. This is the plain rule in all climates and in all localities. After the patient has convalesced, or is in such condition that he can take nutriment, *corn meal* will be found a specific against the poison. This matter I have thoroughly tested, and I can proclaim it as a certainty, that the hydrated peroxide of iron is not a more certain cure for poisoning by arsenic, than that corn-meal is an anti-periodic and specific for the terrible disease known as milk sickness or, 'trembles'. Not only in man is it a specific, but in cattle the cure is certain. Feed either party with it in plenty, give them all they can possibly eat give it as mush and milk, as corn bread, as raw corn meal, or any other manner you can, but give corn to cure milk sickness.—*Cleaveland Medical Gaz.*, from *Amer. Med. Monthly.*

